# Appropriate indications for laparoscopic repeat hepatectomy

**DOI:** 10.1186/s12893-023-02208-y

**Published:** 2023-10-24

**Authors:** Takashi Masuda, Yuichi Endo, Shota Amano, Masahiro Kawamura, Atsuro Fujinaga, Takahide Kawasaki, Yoko Kawano, Teijiro Hirashita, Masayuki Ohta, Masafumi Inomata

**Affiliations:** Department of Gastroenterological and Pediatric Surgery, Oita University Faculty of Medicine, Idaigaoka 1-1, Hasama-machi, Oita, 879-5593 Japan

**Keywords:** Laparoscopic repeat hepatectomy, Repeat hepatectomy, Conversion, HALS, Difficulty score

## Abstract

**Background:**

Laparoscopic repeat hepatectomy (LRH) has increased, but appropriate indications for LRH are unclear. This study aimed to clarify appropriate indications for LRH.

**Methods:**

We retrospectively compared surgical outcomes between open RH (ORH) (n = 57) and LRH (n = 40) groups. To detect difficult cases of complete pure LRH, we examined patients with unplanned intraoperative hand-assisted laparoscopic surgery (HALS)/open conversion (n = 6).

**Results:**

In the LRH versus ORH group, as previous hepatectomy, laparoscopic (75% vs. 12%, p < 0.001) and partial hepatectomy (Hr0) (73% vs. 37%, p = 0.002) were more frequently performed, and as RH procedure, partial hepatectomy (Hr0) (88% vs. 47%, p = 0.0002) was more frequently performed. S1 tumor cases were higher in ORH group (11% vs. 0%), but S2-6 cases were higher in LRH group (73% vs. 49%) (p = 0.02). In LRH group, compared to the pure LRH patients, HALS/open conversion patients underwent significantly more previous hepatectomy with more than lobectomy (Hr2-3) (33% vs. 2.9%, p = 0.033) and more RH procedures with segmentectomy (HrS) (33% vs. 2.9%, p = 0.03). All LRH requiring a repeat hepatic hilar approach were HALS conversions.

**Conclusion:**

Appropriate indications for LRH were previous hepatectomy was laparoscopic partial hepatectomy (Hr0), and RH procedure was partial hepatectomy (Hr0) for S2-6 tumor location. When RH is more than segmentectomy (HrS) requiring a repeat hepatic hilar approach, planned HALS or ORH may be a better approach than pure LRH.

**Supplementary Information:**

The online version contains supplementary material available at 10.1186/s12893-023-02208-y.

## Introduction

Hepatocellular carcinoma and colorectal liver metastasis frequently recur after hepatectomy, but repeat hepatectomy (RH) is aggressively performed because a relatively good prognosis can be expected with RH [1–4]. The first case of laparoscopic liver resection (LLR) was reported in 1991 by Reich et al. [5] With the advancement of laparoscopic techniques and equipment, the rate of LLR has been rapidly increasing worldwide [6].

LLR is still considered a highly difficult procedure, and there are several difficulty scoring systems that classify its levels of difficulty [7–9]. Surgeons are required to select LLR according to the difficulty level of LLR and their surgical skills. Pure LLRs are sometimes difficult to complete, and there are cases in which unplanned intraoperative conversion to hand-assisted laparoscopic surgery (HALS) or open hepatectomy are necessary [10].

Recently, the number of cases of LLR has been increasing even for RH, and the efficacy of laparoscopic RH (LRH) has been reported [11–15]. However, RH is more difficult than the initial hepatectomy due to adhesions, deformity of the liver, and displacement of the vessels. The indications for LRH are not clear, and the choice of open or laparoscopic is made at the discretion of each institution and surgeon. It is still unclear which cases are appropriate for LRH.

The aim of this study was to retrospectively investigate the characteristics of open and LRH cases in our department and to clarify the appropriate indications for LRH.

## Materials and methods

### Patients and methods

In total, 97 patients underwent RH at the Department of Gastroenterological and Pediatric Surgery, Oita University Faculty of Medicine between January 2010 to November 2021. Among them, 57 patients underwent open RH (ORH) and 40 patients underwent LRH. There were no planned HALS cases in the LRH group. We performed a pure laparoscopic approach first, and then converted to HALS or open surgery in difficult cases. The operator was different in each case although the surgical team included expert surgeons who were board-certified by the Japanese Society of Hepato-Biliary-Pancreatic Surgery and/or the Japan Society for Endoscopic Surgery. The use of anti-adhesive agents was initiated at our institution since November 2010 for open hepatectomy and since August 2019 for laparoscopic hepatectomy. We collected information on age, sex, body mass index (BMI), details of previous hepatectomy, the use of anti-adhesive agents, tumor characteristics, difficulty score (DS) for laparoscopic hepatectomy [9], clinical data, histopathology, surgical methods, operative time, blood loss, length of postoperative hospital stay, and postoperative complications from the patients’ medical records. We retrospectively compared these data between the ORH and LRH groups to identify appropriate indications for LRH. Furthermore, the LRH group was stratified by factors of the previous hepatectomy to investigate its influence on the LRH. In this study, we considered unplanned intraoperative HALS and open conversion cases to be difficult for LRH. To detect difficult cases of complete pure LRH, we also further examined the patients receiving HALS/open conversion.

This study was approved by the Ethics Committee of Oita University Faculty of Medicine (No. 1601).

### Definitions

The extent of liver resection was classified using the General Rules for the Clinical and Pathological Study of Primary Liver Cancer by the Liver Cancer Study Group of Japan as follows. Hr0: resection of less than one segment (partial hepatectomy), HrS: resection of one segment (segmentectomy), Hr1: resection of one section (sectionectomy), Hr2: resection of two sections (lobectomy and central bisectionectomy), Hr3: resection of three sections [16]. To perform segmentectomy (HrS), there are two main approaches to the responsible Glissonean pedicle either from the hilum of the liver (the hilar approach) or from the liver surface (the intrahepatic approach) [17]. The anatomical hepatic resections other than S7 and S8 segmentectomy were basically performed by the hilar approach; because the roots of the Gllissonean pedicles for S7 and S8 locate a couple of centimeters deeper from the hilum, S7 and S8 segmentectomies were performed by the intrahepatic approach. Even in cases where the hilar approach was performed in the previous hepatectomy, if more than segmentectomy (HrS) was necessary for RH, we performed a repeat access to the Gllisonean hilum to control blood inflow (a repeat hepatic hilar approach).

To determine the safety indications for LLR, a difficulty scoring system for LLR is useful [7–9]. In this study, we used the difficulty scoring system of the IWATE criteria [9], which is defined by the extent of the liver resection, tumor location, tumor size, liver function, HALS/Hybrid, and tumor proximity to major vessels. Important postoperative complications were defined as those with a Clavien-Dindo classification grade of III or more [18].

### Surgical procedure of HALS and open conversion

Ther were two types of surgical procedure of intraoperative HALS conversion, which differed depending on the surgeon. In the first type, the surgeon stood on the right side of the patient to allow assistance by the surgeon’s own left hand though a small 7-cm incision in the right subcostal abdomen. In the second type, the surgeon stood on the left side of the patient to allow assistance by the surgeon’s own left hand though a small 7-cm incision in the middle of the upper abdomen. Open conversion was performed via a J-shaped incision.

### Statistical analysis

Continuous data are expressed as the mean ± standard deviation, and categorical data are expressed as counts, with the associated percentile value calculated. The Student *t*-test was used to compare continuous data, and Pearson’s χ^2^ test was used for categorical data. A p value < 0.05 was considered to indicate statistical significance. All statistical analyses were performed with JMP software version 14.2 for Windows (SAS Institute, Cary, NC, USA).

## Results

### Comparison of patient clinical characteristics (ORH vs. LRH)

The patients’ clinical characteristics can be compared between the ORH and LRH groups in Table 1. Regarding tumor location, S1 cases were more frequent in the ORH group (11% vs. 0%), whereas S2-6 cases were more frequent in the LRH group (73% vs. 49%) (p = 0.02). As the previous hepatectomy, open hepatectomy was performed significantly more frequently in the ORH group (88% vs. 25%, p < 0.001), and laparoscopic hepatectomy was performed significantly more frequently in the LRH group (75% vs. 12%, p < 0.001). Regarding the extent of previous hepatectomy, performance of more than lobectomy (Hr2-3) was significantly more frequent in the ORH group (30% vs. 7.5%, p = 0.002), and partial hepatectomy (Hr0) was more frequent in the LRH group (73% vs. 37%, p = 0.0013). Regarding the relationship of location between RH and the previous hepatectomy, opposite lobe cases were more frequent in the ORH group (51% vs. 12%, p = 0.04), but same lobe cases were more frequent in the LRH group (70% vs. 49%, p = 0.04).

**Table 1:** Patient characteristics in the ORH vs. LHR group

Characteristics	ORH (n = 57)	LRH (n = 40)	P
Sex (male), n (%)	46 (81%)	29 (73%)	0.34
Age, years	68.7 ± 1.4	70.7 ± 1.6	0.36
BMI (kg/m^2^)	23.2 ± 0.4	23.1 ± 0.5	0.77
Diagnosis			0.1
CRLM, n (%)	23 (40%)	14 (34%)	
HCC, n (%)	29 (51%)	26 (65%)	
ICC, n (%)	5 (8.8%)	0 (0%)	
Frequency of hepatectomy (2 times/≥3 times), n (%)	43 (75%)/14 (25%)	30 (75%)/10 (25%)	0.96
Tumor location (S1/S2-6/S7-8), n (%)	6 (11%)*/28 (49%)*/23 (40%)	0*/29 (73%)*/11 (28%)	**0.02**
Maximum tumor size (mm)	26.9 ± 1.7	21.7 ± 2.1	0.06
Number of tumors	2.1 ± 0.2	1.4 ± 0.3	**0.04**
Previous hepatectomy			
Laparoscopy/open, n (%)	7 (12%)/50 (88%)	30 (75%)/10 (25%)	**< 0.001**
Hr0/HrS-1/Hr2-3, n (%)	21 (37%)*/19 (33%)/17 (30%)*	29 (73%)*/8 (20%)/3 (7.5%)*	**0.002**
Location with previous hepatectomy			
Same/opposite lobe, n (%)	28 (49%)/29 (51%)	28 (70%)/12 (30%)	**0.04**
Adjacent/non-adjacent section, n (%)	43 (75%)/14 (25%)	32 (80%)/8 (20%)	0.6

Abbreviations: BMI, body mass index; CRLM, colorectal liver metastasis; HCC, hepatocellular carcinoma; ICC, intrahepatic cholangiocarcinoma; LRH, laparoscopic repeat hepatectomy; ORH, open repeat hepatectomy

* Significant difference by post hoc analysis

### Comparison of surgical outcomes (ORH vs. LRH)

Surgical outcomes between the ORH and LRH groups can be compared in Table 2. For the extent of RH, more than sectionectomy (Hr1-2) was performed significantly more frequently in the ORH group (33% vs. 5%, p = 0.002), whereas partial hepatectomy (Hr0) was performed significantly more frequently in the LRH group (88% vs. 47%, p < 0.001). RH requiring a repeat hepatic hilar approach was more frequently performed in the ORH group (35% vs. 5%, p = 0.0005). Among the surgical outcomes, the ORH group had significantly longer operative time (278.1 ± 13.1 vs. 210.3 ± 15.7 min, p = 0.001), more blood loss (628.5 ± 53.8 vs. 122.6 ± 65.4 mL, p < 0.001), and longer postoperative hospital stay (18.0 ± 1.5 vs. 9.0 ± 1.7 days, p < 0.001). In the LRH group, there were five cases of HALS conversion and one case of open conversion.

**Table 2:** Surgical outcomes in the ORH vs. LRH group

Outcome	ORH (n = 57)	LRH (n = 40)	P
RH (Hr0/HrS/Hr1-2), n (%)	27 (47%)*/11 (19%)/19 (33%)*	35 (88%)*/3 (7.5%)/2 (5%)*	**0.0002**
RH requiring a repeat hepatic hilar approach, n (%)	20 (35%)	2 (5%)	**0.0005**
HALS conversion, n (%)		5 (13%)	
Open conversion, n (%)		1 (2.5%)	
Operative time (min)	278.1 ± 13.1	210.3 ± 15.7	**0.001**
Blood loss (mL)	628.5 ± 53.8	122.6 ± 65.4	**< 0.001**
Complications after surgery (CD ≥ III), n (%)	8 (14%)	1 (2.5%)	0.054
Postoperative hospital death, n (%)	1 (1.8%)	0	
Postoperative hospital stay (days)	18.0 ± 1.5	9.0 ± 1.7	**< 0.001**

Abbreviations: CD, Clavien-Dindo classification; HALS, hand-assisted laparoscopic surgery; LRH, laparoscopic repeat hepatectomy; ORH, open repeat hepatectomy; RH, repeat hepatectomy

* Significant difference by post hoc analysis

### Comparisons of patient’s characteristics in LRH group stratified by previous operative factors

For patients in the LRH group, we performed an additional analysis of factors that might affect surgical outcomes. According to whether the previous hepatectomy was laparoscopic or open, the operative time was significantly longer when the previous hepatectomy was an open hepatectomy (271.7 ± 27.7 vs.189.8 ± 16.0 min, p = 0.01), but this did not affect the postoperative course (supplementary Table S1). When the location of the previous hepatectomy was in the same or opposite lobe, the rate of HALS/open conversion tended to be higher when the location was in the same lobe versus the opposite lobe (18% vs. 8.3%, P = 0.09) (supplementary Table S2), but the difference was not significant.

### Comparisons of patient characteristics of pure LRH vs. HALS/open conversion

Among patients in the LRH group, unplanned intraoperative HALS/open conversion was performed in six patients (supplementary Table S3). We compared the complete pure LRH group with the HALS/open conversion group to examine the risk factors for HALS/open conversion (Table 3). Patients in the HALS/open conversion group had a significantly greater extent of previous hepatectomy with more than lobectomy (Hr2-3) (33% vs. 2.9%, P = 0.033), more RH with segmentectomy (HrS) (33% vs. 2.9%, p = 0.03), more RH requiring a repeat hepatic hilar approach (33% vs. 0%, p = 0.0006), higher IWATE criteria DS (5.3 ± 0.8 vs. 3.5 ± 0.3, p = 0.04), and longer operative time (302.2 ± 35.1 vs. 194.1 ± 14.8 min, p = 0.007). In particular, all cases of RH requiring a repeat hepatic hilar approach (2 of 2 cases) ended up as HALS conversions (Table 3, supplementary Table S3). Although there was no significant difference, in terms of the relationship with the previous hepatectomy, 83% of HALS/open conversion procedures were performed in the same lobe, and 100% were performed in adjacent sections.

**Table 3:** Characteristics of patients with pure LRH vs. HALS/open conversion

Characteristic	Pure LRH (n = 34)	HALS/open conversion (n = 6)	P
Sex (male), n (%)	26 (76%)	3 (50%)	0.18
Age (years)	71.3 ± 1.9	67.3 ± 4.5	0.43
BMI (kg/m^2^)	22.8 ± 0.5	24.6 ± 1.2	0.17
Diagnosis			0.4
CRLM, n (%)	11 (32%)	3 (50%)	
HCC, n (%)	23 (68%)	3 (50%)	
Frequency of hepatectomy (2 times/≥3 times), n (%)	27 (79%)/7 (21%)	3 (50%)/3 (50%)	0.13
Tumor location (S2-6/S7-8), n (%)	26 (76%)/8 (24%)	3 (50%)/3 (50%)	0.18
Maximum tumor size (mm)	20.7 ± 1.5	26.8 ± 3.5	0.11
Number of tumors	1.3 ± 0.1	1.8 ± 0.2	**0.01**
Previous hepatectomy			
Laparoscopy/open, n (%)	27 (79%)/7 (21%)	3 (50%)/3 (50%)	0.13
Extent of resection (Hr0/HrS-1/Hr2-3), n (%)	26 (76%)/7 (21%)/1 (2.9%)*	3 (50%)/1 (17%)/2 (33%)*	**0.033**
The use of anti-adhesive agents, n (%)	5 (15%)	1 (17%)	0.9
Relationship to previous hepatectomy			
Same/opposite lobe, n (%)	23 (68%)/11 (32%)	5 (83%)/1 (17%)	0.44
Adjacent/non-adjacent section, n (%)	26 (76%)/8 (24%)	6 (100%)/0 (0%)	0.18
RH (Hr0/HrS/Hr1-2), n (%)	31 (91%)/1 (2.9%)*/2 (5.9%)	4 (67%)/2 (33%)*/0	**0.03**
RH requiring a repeat hepatic hilar approach, n (%)	0	2 (33%)	**0.0006**
Tumor proximity to major vessels, n (%)	1(2.9%)	0	0.67
IWATE criteria difficulty score	3.5 ± 0.3	5.3 ± 0.8	**0.04**
Operative time (min)	194.1 ± 14.8	302.2 ± 35.1	**0.007**
Blood loss (mL)	97.3 ± 34.9	261.7 ± 81.9	0.07
Complications after surgery (CD ≥ III), n (%)	1 (2.9%)	0	0.67
Postoperative hospital stay (days)	8.9 ± 0.6	9.0 ± 1.4	0.97

Abbreviations: BMI, body mass index; CD, Clavien-Dindo classification; CRLM, colorectal liver metastasis; HALS, hand-assisted laparoscopic surgery; HCC, hepatocellular carcinoma; LRH, laparoscopic repeat hepatectomy; RH, repeat hepatectomy

* Significant difference by post hoc analysis

## Discussion

In this study, we retrospectively reviewed cases of ORH and LRH performed in our department to determine which cases were appropriate for LRH. Comparing the ORH group to the LRH group, open and more than lobectomy (Hr2-3) were more frequently performed as the previous hepatectomy, and more than sectionectomy (Hr1-2) was more frequently performed for RH. RH requiring a repeat hepatic hilar approach was more frequently performed in the ORH group. In the LRH group, however, laparoscopic and partial hepatectomy (Hr0) were more frequently performed as the previous hepatectomy, and partial hepatectomy (Hr0) was more frequently performed for RH. The number of S1 cases was higher in the ORH group, and that of S2-6 cases was higher in the LRH group. When we compared the complete pure LRH group to the HALS/open conversion group to examine the risk factors for unplanned intraoperative HALS/open conversion, the HALS/open conversion group had a significantly greater extent of previous hepatectomy with more than lobectomy (Hr2-3), more RH with segmentectomy (HrS), and more RH requiring a repeat hepatic hilar approach. In particular, all cases of RH requiring a repeat hepatic hilar approach ended up as HALS conversions.

RH is more difficult than the initial hepatectomy due to adhesions, deformity of the liver, and displacement of the vascular vessels. The degree of adhesions present during RH has been reported to be related to the difficulty of the surgery and to correlate with postoperative complications [19, 20]. Some reports comparing LRH with ORH have shown the benefit of LRH [12, 13, 21]. In particular, LRH has been reported to reduce blood loss, with the same incidence of postoperative complications and a shorter postoperative hospital stay compared to those of ORH [13, 21]. However, there are few reports describing the cases that are suitable for LRH. Kinoshita et al. examined the difficulty classification of LRH [11, 22]. They reported five preoperative predictive factors for difficult LRH as follows: a history of previous open liver resection, that of two or more previous liver resections, that of a previous major liver resection (not less than sectionectomy), tumor near the resected site of the previous liver resection, and intermediate or high difficulty as indicated by the difficulty scoring system [7]. When 0 to 3 of these factors were present, the patient was in the low or intermediate difficulty class and the indication for LRH was considered to be good. In contrast, when 4 or 5 of these factors were present, the patient was in the high difficulty class and could not be recommended for LRH. Our study is more practical, as it refers to specific operative procedures, and is novel in that we refer to the risk factors for HALS/open conversion and the cases in which planned HALS or ORH may be a better approach than pure LRH.

In our study, cases with tumor locations of S2-6 were more common in the LRH group. For tumors in these location, the difficulty level is relatively low, suggesting a good indication for laparoscopic hepatic resection, and even for LRH [7, 9]. If the previous hepatectomy was an open hepatectomy, the operative time could be prolonged. Cioffi et al. reported that when the initial hepatectomy was an open hepatectomy, compared to laparoscopic hepatectomy, postoperative adhesions at the time of RH were severe and the operative time was longer [23]. In the present study, we considered HALS/open conversion cases to be difficult for LRH, and we compared each factor between the HALS/open conversion group and pure the LRH group to investigate the factors causing difficult LRH. The relationship between previous hepatectomy and RH was reviewed in terms of the same or opposite lobe and adjacent or nonadjacent section. Although there were no significant differences, 5 of the 6 patients in the HALS/open conversion group had the procedure on the same lobe, and all 6 had adjacent section relationships. We believed this result was reasonable as it also matches the risk factors of previous reports [11, 22]. However, in our study, the number of same lobe cases was higher in the LRH group than in the ORH group, suggesting that LRH is not necessarily inappropriate and that partial hepatectomy (Hr0) would be sufficient. In cases in which the previous hepatectomy was more than lobectomy (Hr2), or in which the RH was more than segmentectomy (HrS), the surgery should be performed with consideration of the possibility of performing intraoperative HALS/open conversion. In particular, in the two patients in whom the previous hepatectomy required a hepatic hilar approach and RH was more than segmentectomy (HrS) requiring a repeat hepatic hilar approach, both patients ended up undergoing HALS conversion, although ORH was more frequently selected for these cases. After hepatectomy with a hepatic hilar approach, adhesions develop in the hepatic hilum, and deformity of the hepatic hilum and displacement of the vessels occur as a result of liver regeneration. The RH requiring a repeat hilar approach becomes more difficult as a result of both of these factors. These cases may correspond to the intermediate class reported by Kinoshita et al. [11, 22]; clinically, however, they are very difficult and should be considered as cases with high-level difficulty. Therefore, we believe that cases requiring a repeat hilar approach for more than segmentectomy (HrS) should be considered for planned HALS or ORH rather than pure LRH at this time.

The present study has several limitations. First, this is a single-center study with a small number of cases. Second, the operator was different in each case although the surgical team included expert surgeons who were board-certified. Third, the stratified factors used in the present study were those generally considered to influence hepatectomy, and not all factors were considered. Fourth, long-term prognoses were not comparable because of the variety of diseases included in this study.

In conclusion, appropriate indications for LRH were as follows: the previous hepatectomy was a laparoscopic partial hepatectomy (Hr0), and the RH procedure was partial hepatectomy (Hr0) for a tumor located at S2-6. In cases in which the previous hepatectomy was more than lobectomy (Hr2), or when the RH procedure was more than segmentectomy (HrS), the surgery should be performed with consideration of the possibility of performing intraoperative HALS/open conversion. Finally, in cases in which the previous hepatectomy required a hepatic hilar approach and RH was more than segmentectomy (HrS) requiring a repeat hepatic hilar approach, planned HALS or ORH may be a better approach than pure LRH.

### Electronic supplementary material

Below is the link to the electronic supplementary material.

Supplementary Material 1

Supplementary Material 2

Supplementary Material 3

## Data Availability

The datasets used and/or analyzed during the current study are available from the corresponding author on reasonable request.
