# Supplementary material for: Appropriate indications for laparoscopic repeat hepatectomy
Source: BMC Surg. 2023 Oct 24;23:322. doi: 10.1186/s12893-023-02208-y (PMC10594730; doi:10.1186/s12893-023-02208-y)
Supplement: Supplementary file 1 — Supplementary Material 1 [file 12893_2023_2208_MOESM1_ESM.docx]

| Supplementary Table S1 Influence of previous hepatectomy (laparoscopic vs. open) on LRH | | | |
| --- | --- | --- | --- |
|  | Previous hepatectomy | |  |
| Factor | Laparoscopic (n=30) | Open (n=10) | P |
| Tumor location (S2-6/S7-8), n (%) | 22 (73%)/8 (27%) | 7 (70%)/3 (30%) | 0.84 |
| Maximum tumor size (mm) | 21.0±1.6 | 23.7±2.8 | 0.4 |
| Number of tumors | 1.2±0.1 | 1.6±0.2 | 0.09 |
| RH (Hr0/HrS/Hr1-2), n (%) | 26 (87%)/2 (6.7%)/2 (6.7%) | 9 (90%)/1 (10%)/0 | 0.67 |
| IWATE criteria difficulty score | 3.8±0.4 | 3.6±0.6 | 0,76 |
| Operative time (min) | 189.8±16.0 | 271.7±27.7 | **0.01** |
| Blood loss (mL) | 110.0±38.7 | 159.0±65.9 | 0.53 |
| HALS/open conversion, n (%) | 3 (10%) | 3 (30%) | 0.13 |
| Complications after surgery (CD ≥III), n(%) | 1 (3.3%) | 0 | 0.56 |
| Postoperative hospital stay (days) | 9.3±0.6 | 8.0±1.1 | 0.33 |
| Abbreviations: CD, Clavien-Dindo classification; HALS, hand-assisted laparoscopic surgery; RH, repeat hepatectomy. | | | |
