# Supplementary material for: Appropriate indications for laparoscopic repeat hepatectomy
Source: BMC Surg. 2023 Oct 24;23:322. doi: 10.1186/s12893-023-02208-y (PMC10594730; doi:10.1186/s12893-023-02208-y)
Supplement: Supplementary file 2 — Supplementary Material 2 [file 12893_2023_2208_MOESM2_ESM.docx]

| Supplementary Table S2 Influence of location of previous hepatectomy (same lobe vs opposite lobe) in LRH | | | | |
| --- | --- | --- | --- | --- |
|  | Previous hepatectomy | | |  |
| Factor | Same lobe (n=28) | Opposite lobe (n=12) | | P |
| Maximum tumor size (mm) | 21.8±1.7 | 21.3±2.5 | | 0.88 |
| Number of tumors | 1.4±0.1 | 1.3±0.2 | | 0.9 |
| RH (Hr0/HrS/Hr1), n (%) | 26 (93%)/2 (7.1%)/0 | 9 (75%)/1 (8.3%)/2 (17%) | | 0.08 |
| IWATE criteria difficulty score | 3.9±0.4 | 3.5±0.6 | | 0.58 |
| Operative time (min) | 219.4±17.7 | 189±27 | | 0.35 |
| Blood loss (mL) | 149.1±38.8 | 55±61.8 | | 0.21 |
| HALS/open conversion, n (%) | 5 (18%) | 1 (8.3%) | | 0.09 |
| Complications after surgery (CD ≥III), n (%) | 0 | 1 (8.3%) | | 0.12 |
| Postoperative hospital stay (days) | 8.3±0.6 | | 10.4±1.0 | 0.08 |
| Abbreviations: CD, Clavien-Dindo classification; HALS, hand-assisted laparoscopic surgery; LRH, laparoscopic repeat hepatectomy; RH, repeat hepatectomy. | | | | |
