# Supplementary material for: Appropriate indications for laparoscopic repeat hepatectomy
Source: BMC Surg. 2023 Oct 24;23:322. doi: 10.1186/s12893-023-02208-y (PMC10594730; doi:10.1186/s12893-023-02208-y)
Supplement: Supplementary file 3 — Supplementary Material 3 [file 12893_2023_2208_MOESM3_ESM.docx]

| Supplementary Table S3 Cases of unplanned intraoperative HALS/open conversion | | | | | | | | | | | | | | | |  |
| --- | --- | --- | --- | --- | --- | --- | --- | --- | --- | --- | --- | --- | --- | --- | --- | --- |
| Case | Age (yrs) | | Sex | BMI (kg/m^2^) | History of abdominal surgery or  previous hepatectomy | The use of anti-adhesive agents | Diagnosis | Tumor  location | Tumor  size (mm) | IWATE criteria  DS | Liver cirrhosis | RH surgical procedure | RH requiring a repeat hepatic hilar approach | Conversion | Cause of HALS conversion | |
| 1 | 78 | | Male | 25.8 | Right hemicolectomy Open S6 partial hepatectomy (twice) | - | HCC | S8, S6 | 28 | 5 | - | S6, S8 partial hepatectomy | No | HALS | Severe adhesions around the liver | |
| 2 | 59 | | Female | 25 | Lap-sigmoid colectomy Lap-right hepatectomy | - | CRLM | S4 | 40 | 7 | - | S4 subsegmentectomy | Yes | HALS | Severe adhesions around the liver | |
| 3 | 52 | | Female | 25.8 | Lap-right hemicolectomy Lap-left hepatectomy and S7 partial hepatectomy | - | CRLM | S5, S7 | 38 | 7 | - | S5, S7 partial hepatectomy | No | HALS | Severe adhesions around the liver | |
| 4 | 78 | | Female | 25.1 | Distal gastrectomy cholecystectomy Open S8 partial hepatectomy (twice) | - | Liver metastasis  of GIST | S6 | 20 | 2 | - | S6 partial hepatectomy | No | HALS | Adhesions around liver hilum | |
| 5 | 70 | | Male | 25 | Open anterior sectionectomy | + | HCC | S6 | 25 | 6 | + | S6 segmentectomy | Yes | HALS | Adhesions around liver hilum | |
| 6 | 67 | | Male | 20.8 | Lap-S8 segmentectomy  Lap-S6 partial hepatectomy | - | HCC | S7 | 10 | 6 | + | S7 partial hepatectomy | No | HALS  ⇨open | Poor detection of lesion | |
|  | | Abbreviations: BMI, body mass index; Lap, laparoscopic; CRLM, colorectal liver metastasis; DS, difficulty score; HCC, hepatocellular carcinoma; GIST, gastrointestinal stromal tumor; HALS, hand-assisted laparoscopic surgery; RH, repeat hepatectomy. | | | | | | | | | | | | | | |
